# Structure-programmable solid electrolytes for stack-pressure-free all-solid-state micro-batteries

**DOI:** 10.1093/nsr/nwag345

**Published:** 2026-06-04

**Authors:** Zhijun Yang, Jiaxin Ma, Zhong-Shuai Wu

**Affiliations:** State Key Laboratory of Catalysis, Dalian Institute of Chemical Physics, Chinese Academy of Sciences, China; Center of Materials Science and Optoelectronics Engineering, University of Chinese Academy of Sciences, China; State Key Laboratory of Catalysis, Dalian Institute of Chemical Physics, Chinese Academy of Sciences, China; School of Materials Science and Engineering, Zhengzhou University, China; State Key Laboratory of Catalysis, Dalian Institute of Chemical Physics, Chinese Academy of Sciences, China; Center of Materials Science and Optoelectronics Engineering, University of Chinese Academy of Sciences, China

## Abstract

Structural programming enables the rational construction of solid electrolytes into architected frameworks capable of stress regulation, directional ion transport modulation, and interfacial stabilization, thereby allowing stack-pressure-free operation of all-solid-state microbatteries integrated within microsystems.

The rapid expansion of autonomous microscale systems ranging from microrobots and implantable electronics to distributed sensors strictly demands integrated micropower sources that couple robust long-term stability with intrinsic safety [1]. All-solid-state micro-batteries with customizable configurations emerge as a promising candidate owing to their leakage-free operation and inherent compatibility with miniaturized device architectures. Nevertheless, the reliable operation of solid-state batteries conventionally relies on externally applied stack pressure to maintain intimate interfacial contact and accommodate interfacial deformation. In microscale systems, stringent spatial and structural constraints preclude the application of mechanical pressure, as micro-batteries are inherently required to function as self-supported or load-bearing components. The absence of stack pressure thus renders solid–solid interfaces vulnerable to electro–chemo–mechanical instability. Under zero-stack-pressure conditions, merely tuning intrinsic material properties lacks the mechanical driving force to spontaneously establish conformal contact across rigid micro-interfaces, and is therefore unable to maintain continuous interfacial contact as the lithium anode during stripping [2]. Overcoming this static and dynamic interfacial bottleneck requires a shift toward structural engineering. Crucially, rationally designed electrolyte architectures can effectively internalize stack pressure, leveraging geometric constraints and built-in elastic buffers to autonomously accommodate volumetric fluctuations and sustain intimate contact without external intervention, as confirmed by a rationally designed lamellar composite that integrates two-dimensional superionic sulfide nanosheets with a polymer matrix for stack-pressure-free solid-state batteries [3]. Therefore, solid electrolytes should be regarded not only as ion-conducting media but also as structural frameworks that govern the mechanical and electrochemical behavior of all-solid-state micro-batteries. By deliberately designing the three-dimensional (3D) architecture of the electrolyte, it becomes possible to regulate stress distribution, direct ion transport pathways, and spatially confine interfacial reactions within the device [4].

This perspective highlights the concept of structure-programmable solid electrolytes as a transformative shift beyond conventional passive planar separators. Specifically, structure-programmable architectures are engineered with precise geometric, mechanical, and chemical parameters, enabling adaptive functionality and dynamic responsiveness during battery operation. By integrating mesoscale geometric topologies, spatially graded heterostructures, highly anisotropic alignments, and nanoscale interfacial textures, these programmed elements are deterministically engineered to synergistically regulate the dynamic electro–chemo–mechanical coupling within all-solid-state micro-batteries (Fig. 1). In particular, there are three core programmable interventions: (i) redistributing mechanical stresses to ensure structural compliance under zero-stack-pressure conditions; (ii) rectifying ionic pathways to maximize directional ion flux; and (iii) modulation of interfacial reactions to guide interface evolution toward a metastable equilibrium.

**Figure 1.**
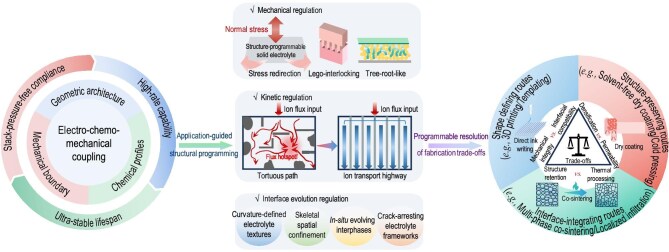
The design framework of structure-programmable solid electrolytes. The paradigm conceptually bridges fundamental electro–chemo–mechanical coupling with deterministic structural inputs (geometric, mechanical, and chemical boundaries) to satisfy macroscopic application demands (left). Through application-guided programming, these static designs are translated into three core functions, including mechanical, kinetic, and interface evolution regulations (middle). Ultimately, this programmable architecture facilitates the resolution of inherent fabrication trade-offs via synergistic manufacturing routes (right).

One central function of structure-programmable solid electrolytes is to reconcile the intrinsic mechanical incompatibility at solid–solid interfaces, arising from the mismatch between the dynamic volumetric deformation of the electrode and the static elastic modulus of the solid electrolyte. In the absence of stack pressure, this mismatch directly leads to contact loss and void formation. Programmable architectures address this limitation by regulating stress distribution rather than relying solely on intrinsic material softening. Geometric designs such as interlocking modular domains [5], tile-and-grout arrays [6], or bio-inspired root-like channels geometrically redistribute localized normal stresses into more delocalized shear components. To prevent shear stresses from inducing interfacial delamination, rationally integrated compliant polymer phases are selectively incorporated as confined mechanical buffers within designated non-critical domains, where they elastically dissipate strain energy. Concurrently, a continuous rigid 3D inorganic skeleton maintains structural integrity and provides an effective barrier against dendrite propagation. The combination of geometric stress redistribution and spatially decoupled elastic buffers acts as an integrated flexible constraint that reversibly accommodates volumetric expansion during plating while preserving conformal contact during stripping, thus enabling stack-pressure-free operation. When scaled to macroscopic systems or high-expansion chemistries, the paradigm translates to low-pressure regimes, utilizing programmed designs to facilitate localized viscoplastic stress relaxation and prevent interfacial delamination.

The sustained physical contact guaranteed by the aforementioned mechanical regulation serves as the indispensable prerequisite for uninterrupted ion transport across the solid–solid interfaces. However, establishing a stable interface alone is insufficient to achieve high-rate operation. A key challenge remains the discrepancy between intrinsic material ionic conductivity (*σ*) and device-level ion flux (*J*). To bridge this gap, we propose the concept of ion-flux-guided electrolyte architecture, wherein transport pathways are spatially engineered to maximize effective ion throughput. Specifically, asymmetric designs (bilayer/multilayer heterostructures) [5] and gradient architectures (gradient porosity) enable rational spatial allocation and smooth kinetic transitions [7]. Furthermore, crystallographic texture engineering (columnar grains) and mesoscale anisotropic architectures (vertically aligned nanosheet arrays) [3] could establish vertically oriented fast-channels. These structural features systematically align ion diffusion paths, suppress lateral ion congestion, and preserve high skeletal densification to minimize resistive boundaries. Compared with traditional homogeneous designs, such vertically aligned architectures have been reported to achieve room-temperature ionic conductivity exceeding 10^−2^ S cm^−1^ and maintain a high critical current density of 5.0 mA cm^−2^ under pressure-less conditions [3]. Consequently, the effective ion diffusion distance (*L*_eff_) is substantially reduced; geometric tortuosity approaches unity (*τ*→1), thereby ensuring that high intrinsic conductivity of electrolyte materials is efficiently translated into a maximized and directional ion flux within all-solid-state micro-batteries.

Fundamentally supported by the physical confinement and structural anchoring of a stable mechanical framework, along with the homogenized internal electric fields created by the uniform ion flux, these structural designs eliminate the intrinsic mechanical incompatibility and current hotspots that cause spontaneous interfacial failure. This in turn empowers the third pillar of modulation of interfacial reactions to guide interface evolution at the nanoscale. Without stack pressure, uncontrolled interphase growth and localized deposition can rapidly amplify interfacial instability. Conventional systems are inherently governed by the spontaneous and often uncontrolled formation of interphases, such as solid electrolyte interphase (SEI) and cathode electrolyte interphase (CEI). In contrast, structure-programmable architectures enable deterministic regulation of interfacial evolution through coupled physical confinement and chemical programming. Engineered concave microcavities and negative-curvature textures homogenize local electric fields, thereby promoting uniform nucleation and suppressing the formation of dendrite-triggering hotspots [8,9]. Simultaneously, these rigid 3D frameworks impose spatial confinement, acting as volumetric buffers that restrict metal deposition and parasitic SEI and CEI growth [5,6]. Beyond physical confinement, these architectures also program interfacial chemistry. Reactive surface layers are engineered into multilayer heterostructures that spatially differentiate dense passivation regions from compliant, stress-buffering domains [7]. Upon attempted penetration by dendrites, sequentially designed interlayers trigger localized physicochemical decomposition. Crucially, the constrained volume expansion of these reaction products generates a mechanical anchoring effect, robustly arresting crack propagation and physically confining microscopic fractures [10]. Unlike conventional planar separators that typically suffer from rapid impedance growth, such rationally engineered interphases can substantially reduce interfacial resistance [7], thereby supporting ultra-stable operation over 10 000 cycles [10]. Ultimately, through the coupled regulation of interfacial physics and chemistry, these structural designs transform the solid electrolyte into an active dynamic interfacial modulator, directing the evolution of SEI and CEI dynamics toward a stable equilibrium.

Translating these blueprints into practical devices via advanced manufacturing (3D printing, co-sintering) is fundamentally challenged by the complex coupling of geometry, materials, and thermodynamics. However, our programmable framework intrinsically resolves these interdependent constraints (Fig. 1). First, the thermal conditions required for densification inevitably induce viscous flow, threatening the structural fidelity of complex 3D architectures. Programmable geometries neutralize this thermodynamic sintering tension by fragmenting continuous macroscopic shrinkage into isolated micro-strains, thereby preserving topological fidelity. Simultaneously, a fundamental densification-permeability paradox emerges: a dense electrolyte framework is required for efficient ion transport, whereas an interconnected porous network is indispensable for effective electrode infiltration. Critically, limited permeability fundamentally constrains the mass loading of active materials. The ion-flux-guided architectures bridge this gap by utilizing vertically aligned fast-channels to physically decouple bulk densification from interfacial wetting. Furthermore, increasing porosity to facilitate transport inherently compromises mechanical stiffness. Programmable architectures overcome this porosity-modulus dilemma via intrinsic stress redistribution, utilizing interlocking domains to translate localized normal stresses into less damaging shear components. Ultimately, resolving these coupled constraints bridges the gap between structural design and device integration, advancing the paradigm toward application-directed structural strategies.

The paradigm of structure-programmable solid electrolytes represents a fundamental shift from passive material optimization to the active spatiotemporal management of complex electro–chemo–mechanical processes. When integrated with advanced fabrication methodologies, these principles enable electrolyte architectures that function not only as ionic conductors but also as structural frameworks governing device stability and performance. To propel this field beyond static structural regulation toward intelligent and adaptive electrolyte systems, the exploration of this expansive multiscale design space will increasingly rely on AI-assisted inverse design and digital twin frameworks. By coupling multiphysics simulations with generative models and Bayesian optimization, these tools can directly optimize explicit features (for example, interlocking topologies, porosity gradients) and extract latent structural parameters, ultimately balancing theoretical performance limits with practical manufacturing feasibility. Together, these advances will pave the way for monolithic, structural, stack-pressure-free all-solid-state micro-batteries, transforming micropower sources from mere energy-storage units into integrated and load-bearing components that fundamentally empower next-generation autonomous microsystems.

## References

[1] Ma J, Lee SY, Wu ZS. Natl Sci Rev 2025; 12: nwaf467. 10.1093/nsr/nwaf467 41346932 PMC12673571

[2] Li Q, Chen L, Jiao J et al. Natl Sci Rev 2026; 13: nwaf540. 10.1093/nsr/nwaf540 41608039 PMC12839520

[3] Lan X, Li Z, Zhao C et al. Nat Nanotechnol 2026; 21: 388–96. 10.1038/s41565-025-02106-9 41540285 PMC13017535

[4] Li H, Li M, Weng S et al. Natl Sci Rev 2025; 12: nwaf363. 10.1093/nsr/nwaf363 41040487 PMC12485985

[5] Mei Y, Hu X, Wang H et al. Matter 2025; 8: 102469. 10.1016/j.matt.2025.102469

[6] Zhao Q, Cao Z, Wang X et al. J Am Chem Soc 2023; 145: 21242–52. 10.1021/jacs.3c04279 37751194

[7] Wan H, Wang Z, Zhang W et al. Nature 2023; 623: 739–44. 10.1038/s41586-023-06653-w 37880366

[8] Ning Z, Jolly DS, Li G et al. Nat Mater 2021; 20: 1121–9. 10.1038/s41563-021-00967-8 33888903

[9] Wang J, Jia L, Du Y et al. Natl Sci Rev 2026; 13: nwag015. 10.1093/nsr/nwag015 41835225 PMC12988355

[10] Ye L, Li X. Nature 2021; 593: 218–22. 10.1038/s41586-021-03486-3 33981053

